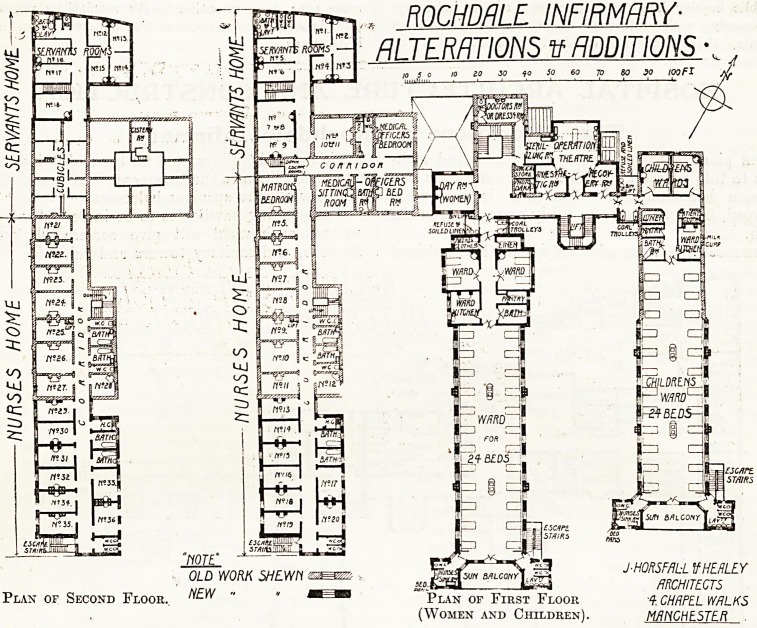# The Alterations at Rochdale Infirmary

**Published:** 1913-11-08

**Authors:** 


					HOSPITAL ARCHITECTURE AND CONSTRUCTION,
The Alterations at Rochdale Infirmary.
. Tile plans show extensive alterations and addi-
"ons to this hospital, which has on more than one
occasion been altered. The site is somewhat
Cl'amped, but appears to be fairly open towards the
south.
The most important addition will be the new ward
pavilion between the nurses' home and the existing
^ard pavilion which also is to be extended. The
frew pavilion will be two storeys in height, and will
?i)Te a total accommodation of 56 beds, together
^vith the usual ward offices. The sanitary offices
are placed in towers at the south end of the pavilion,
arid include a well-arranged sink room and w.c.
A?r nurses on one side, and w.c.s and lavatory for
Patients on the other side. The bathroom is placed
close to the entrance of the ward. The only point
?pen to criticism is the absence of any provision for
emptying bed-pans in connection with the two small
^ards. The pavilion is separated from the main
building ley a cut-off lobby in which provision is
made for coal trolleys, and for the dispatch of refuse
and soiled linen to the basement. At the south end
of the wards are spacious balconies.
The existing pavilion is to be increased in length
so that each ward will give accommodation for
24 beds, and sanitary towers and balconies will be
arranged at the south end in a similar way to those-
of the new pavilion.
Two wards of two beds each will be attached to
this enlarged pavilion, but are placed on the further
side of the main corridor. This arrangement is-
necessitated by existing conditions which could not
be altered.
In the centre between the two pavilions is the
main staircase with an electric lift in the well hole.
To the north of the connecting corridor will be a
complete casualty department, with an entrance and'
exit for ambulances from the street. The casualty
department includes a waiting room, large surgery..
two dressing rooms, an electrical department, and
? com
1 STORE
m tebbuons v BDomoNs
\KITCH?!1\
MTU
5CULLEI
30 90 too FX
kitchen rm
mar on m
_ MULANC\
\ttlS YfilD
HUMt
mms
Dime
HALL
^C0TZ?l
! OFFICE. n ROOM
c/mu
twrv ?|
?Liarna
mCFFKA
fmnm
vmna\
//7?/f? (wj
Mmotis
STORE.
EXISTING
ISOLATION
PAVILION
conniDOR op?n
on SOUTH S/Dt.
R?fU5t*
50;L?D L/HEf
vTTcjal
fflTROLLItS
COAL )
TROLULSi
MJRSE5
Dime
ROOM
NUR5?S1
5rmp
?9=' I q
ipwmc^\
[Wf/W
! SITTING
ROOM
OLD WORK SHEWN THUS
NEW ??
'nurses*
h/rwd
ROOM I
J-MORSFALL V HE/1LEY
ARCHITECTS
? CHAPEL WALKS
MANCHESTER-
LSCAPL
STAIRS
[SUN BfiLCOnr
?sc*rt
I STAIRS
srms
.SUN MLCOtiY\ifF
BCD
fH13
1-48 THE HOSPITAL N OVEMBER 8, 1913".
an emergency ward for noisy or dying cases. Other
alterations on the ground floor include a new day
rooni for male patients, rearrangement of the
kitchen offices, and an addition to the nurses'
home.
On the first floor over the casualty department is
a new theatre suite, including anaesthetic and
recovery rooms, sterilising room, and theatre.
| Additional rooms for nurses and servants on this
| and the floor above complete the work.
The whole scheme has been planned by, and is
being carried out under the supervision of, Mr.
Hugh Healey, of the firm of Horsfall and Healey,
of Manchester.
L5?/?otvj
b_cii_
wcraam
{OKWWM
%DIC/IL ;
Ff/C?R5 I
imOOMl
rm- oKrnidir|[nj[
rw?ffTfaj[ID|
Mw/w | t/rr/Rf I
lOtfll
connidor
"iicJ-S/OT
3/rmcqsffiC] S?D
flOCW 1 flf? w
mw\
(worn)
ItFUStV -
SOILIDLIHV
COAL'
TnOLLrr
[rtoLLcrs
.StilLDKLNy"
IP wm_*
|{-?^B?OS?i|
\?sc/rrt
\srms
5i/n 6Alcony
\?3CAft
\5T/JIR5
SUIV BALCONY
ROCHDALE. INFIRMARY-
ALTERATIONS tf ADDITIONS ?.
- ? ^ j
m mo.
BEDROOM
ROOM
OLD WORK SHEWN
HEW ??
123
Plan of First Floor
(Women and Children).
J-HORSFALL If HE flLEY
ARCHITECTS
A. CHAPEL WALKS
MANCHESTER
a n"l
IS
Plan of Second Floor.

				

## Figures and Tables

**Figure f1:**
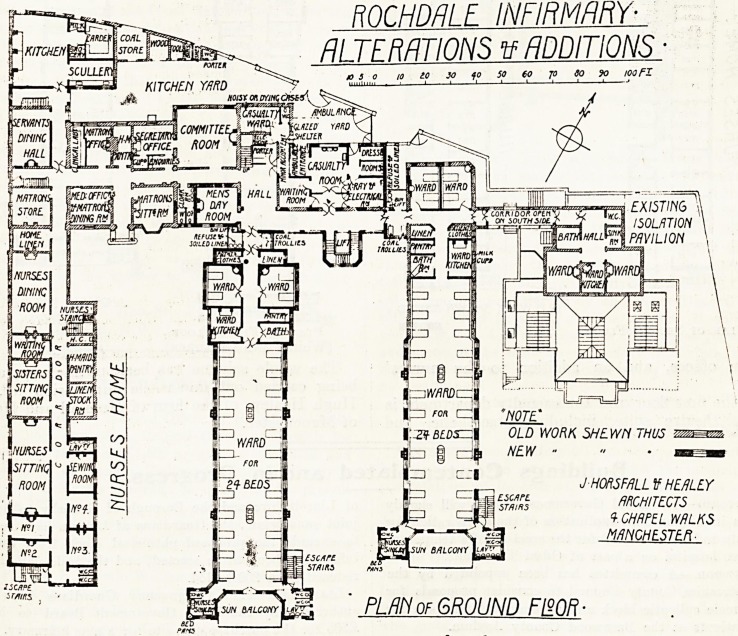


**Figure f2:**